# Effects of Zinc Lactate Supplementation on Growth Performance, Intestinal Morphology, Serum Parameters, and Hepatic Metallothionein of Chinese Yellow-Feathered Broilers

**DOI:** 10.1007/s12011-021-02785-0

**Published:** 2021-06-17

**Authors:** Lina Long, Xichen Zhao, Haojie Li, Xia Yan, Huihua Zhang

**Affiliations:** 1 School of Life Science and Engineering, Foshan University, Foshan, 528231 China; 2 College of Animal Science, South China Agricultural University, Tianhe District, 483Wushan Road, Guangzhou, 510642 China; 3 Institute of Animal Science, State Key Laboratory of Livestock and Poultry Breeding, Guangdong Academy of Agricultural Sciences, Guangzhou, 510640 China

**Keywords:** Zinc lactate, Broiler, Growth performance, Serum parameter, Metallothionein

## Abstract

In poultry, organic zinc compounds have higher bioavailability than inorganic zinc sources. However, as an organic zinc source, the application of zinc lactate (ZL) on Chinese yellow-feathered broilers has been rarely reported. Hence, the present study aimed to investigate the effects of ZL supplementation on growth performance, small intestinal morphology, serum biochemical parameters, immune organ index, as well as hepatic metallothionein of Chinese yellow-feathered broilers. A total of 2100 broilers (19 days old) were randomly assigned to 5 treatment groups, including the control (fed basal diet), ZL40 (basal diet plus 40 mg/kg ZL), ZL60 (basal diet plus 60 mg/kg ZL), ZL80 (basal diet plus 80 mg/kg ZL), and ZS80 (basal diet plus 80 mg/kg ZS. Each treatment group had 6 replicates with 70 chickens per replicate. Compared to the control group, the ZL40 and the ZS80 groups had a lower feed to gain ratio (*P* < 0.05), ZL40 group had higher duodenum and ileum villus heights (*P* < 0.05), and ZS80 and ZL80 groups had a lower ratio of villus height to crypt depth in the jejunum (*P* < 0.01). In addition, the ZL60 group had a higher concentration of total protein (*P* < 0.05) and activity of glutathione peroxidase (GSH-Px) (*P* < 0.01) compared with the ZS80 and the control groups. Interestingly, the ZL40, ZL60, and ZL80 groups all had higher levels of hepatic metallothionein than the other groups (*P* < 0.01). In conclusion, zinc lactate had a higher bioavailability and could be used as an alternative to zinc sulfate.

## Introduction

Zinc (Zn), as one of the trace minerals, serves as a co-factor of hundreds of endogenous enzymes and thousands of transcription factors involved in the various biochemical metabolic events in the body and thus indispensable to animals [1–4]. Zn deficiency can cause pathological changes, decrease growth, reduce immunity, as well as promote the production of free radicals, which lead to oxidative damage to animals [5, 6]. The NRC (1994) [7] recommends 40 mg/kg dietary zinc for commercial broiler chickens to prevent zinc deficiency [8]. Studies showed that higher zinc intake (60 ~ 180 mg/kg) promotes the digestive function of animals including increased activities of digestive enzymes and improved intestinal morphology as well as function [9, 10]. Therefore, the zinc requirement of broilers recommended by NRC (1994) might be outdated. Inorganic zinc (zinc sulfate, zinc oxides, etc.) is mainly used in poultry production to maximize performance [11–13]. Unfortunately, supplementing inorganic zinc may cause environmental pollution due to low bioavailability [14, 15]. In recent years, organic forms of zinc (zinc methionine, zinc glycine, etc.) have been used in poultry production to replace inorganic zinc due to its higher bioavailability [1, 9, 16, 17]. Previous studies demonstrated that dietary supplementation of organic zinc increased weight gain and improved the feed efficiency of broilers [2, 8].

Zinc lactate (ZL) is organic zinc and has been used in animal diets. A previous study revealed that dietary supplementation of zinc lactate (100 mg/kg) significantly improved the small intestinal morphology (increased the villus length and decreased crypt depth), nutrient utilization, and thereby improved the growth performance of pigs [18]. In growing rabbits, zinc lactate supplementation (80 mg/kg) improved the small intestinal morphology and growth performance. Zinc content in the liver was also increased [19]. Studies on IPEC-J2 epithelial cells treated with zinc lactate showed promoted cell proliferation and upregulated metallothionein 1 [20]. However, studies of ZL in the poultry industry have been limited, especially on the slow-growing broilers such as Chinese yellow-feathered broilers. The optimum dosage of zinc lactate in broilers’ diet remains uncertain. Therefore, the present study was carried out to determine the effects of dietary zinc lactate on the growth performance of Chinese yellow feather broilers compared with that of feed-grade zinc sulfate. The optimal dosage of dietary zinc lactate on yellow-feathered broilers would also be evaluated as a reference zinc source in a slow-growing broilers diet.

## Materials and Methods

### Experimental Design, Animals, and Housing

A total of 2100 Chinese yellow-feathered broilers (male, 19 days old) were randomly divided into five groups including the control (basal diet), ZL40 (basal diet plus 40 mg/kg ZL), ZL60 (basal diet plus 60 mg/kg ZL), ZL80 (basal diet plus 80 mg/kg ZL), and ZS80 (basal diet plus 80 mg/kg ZS). Each group had six replicates with 70 chickens per replicate. The experimental diets were formulated according to the NRC (1994) and met the recommended requirements of growing broilers. All broilers had free access to feed and water and were housed in the floor pens (3.5 m × 2.0 m × 1.0 m) under the same environmental conditions. A 23:1 light–dark regime was used throughout the trial. The newly hatched birds were housed in a room that had a temperature of 34 ~ 36 °C, and the temperature was reduced by 3 °C each week until 4 weeks later (28 days old).

### Records, Calculations, and Sampling

The body weight (BW) and feed intake were recorded (TCS-150, 0.05/150 kg, Rongcheng, China) on days 19, 38, and 62 in the morning. The average daily feed intake (ADFI), average daily gain (ADG), and feed to gain ratio (F/G) were calculated according to the values of BW and feed intake during the trial. Approximately 100 g of feed samples were collected, blended, and then stored in a − 20 °C freezer (BCD-321 W, Robert Bosch, Germany) for future analysis of Zn content.

Six broilers were randomly selected from each treatment group on days 38 and 62. Before slaughtering, a ~ 5 ml of blood sample was collected from each chicken after at least 12-h fasting. Blood samples were kept still at room temperature for 2 h and then centrifuged (CT14RD II, Shanghai, China) at 3000 × r for 10 min at room temperature. The serum was collected and stored at − 80 °C freezer (Panasonic MDF-U74V, Japan) until further analysis. After slaughtering, the spleen, thymus, and bursa of Fabricius were separated and weighted (DT200, 0.2/200 g, Brother, America). The organ indices were calculated [21]. Segments of duodenum, jejunum yolk phloem, and ileocecal orifice from broilers on day 62 were collected, rinsed with cold phosphate-buffered saline (PBS), and immobilized in 4% formaldehyde for 24 h. Liver samples were collected and stored at − 80 °C for further analysis.

### Chemical Analysis

The content of Zn in feed samples determined by an atomic absorption spectrometer (AA-7000, SHIMADZU, Japan) [22] (Tables 1 and 2). The serum concentration of albumin (A028-2–1), total protein (A045-4–2), and the enzyme activities of alkaline phosphatase (A059-2–2), glutathione peroxidase (GSH-Px) (A005-1–2), and superoxide dismutase (SOD) (A001-1–2) were determined by using commercial assay kits (Nanjing Jiancheng Bioengineering Institute, China) according to the manufacturer’s instructions.

**Table 1:** Ingredients and composition of the experimental diet (as-fed basis)

Ingredients (%)	Stage		Nutrient levels	Stage	
	19 ~ 37	38 ~ 61		19 ~ 37	38 ~ 61
Corn	63.46	65.46	ME (MJ/kg)	12.54	12.96
Soybean meal	28.00	24.00	CP (%)	18.63	17.60
Salt	0.30	0.30	Lys (%)	0.99	0.92
Limestone	1.41	1.41	Met (%)	0.46	0.42
Monocalcium phosphate	1.33	1.33	Ca (%)	0.88	0.84
Soybean oil	2.50	3.50	AP (%)^2^	0.40	0.38
Corn gluten meal	2.00	3.00	Zinc (ppm)	31	28
Premix^1^	1.00	1.00			
Total	100.0	100.0			

^1^Supplied per kilogram of diet: VA, 6000 IU; VD3, 2000 IU; VE, 30 mg; VK3, 2 mg; VB1, 3 mg; VB2, 5 mg; pantothenic acid, 800 mg; choline chloride, 1500 mg; nicotinic acid, 30 mg; pyridoxine, 3 mg; folic acid, 500 mg; biotin, 0.2 mg; VB12, 1 mg; Fe, 80 mg; Cu, 8 mg; Mn, 80 mg; I, 0.35 mg; Se, 0.3 mg; Co, 0.2 mg

^2^*AP*, available phosphorus

**Table 2:** Concentrations of zinc in the diets (calculated, mg/kg)

Items	ZL40^1^	ZL60^1^	ZL80^1^	ZS80^2^	Control
19 ~ 37 d	71	91	111	111	31
38 ~ 61 d	68	88	108	108	28

^1^Supplemented with zinc lactate on the basal diet, and the additive dosage was 40 mg/kg, 60 mg/kg, 80 mg/kg, respectively; *ZL*, zinc lactate

^2^Supplemented with zinc sulfate on the basal diet, and the additive dosage was 80 mg/kg; *ZS*, zinc sulfate

### Small Intestinal Morphology

Intestinal samples were embedded in paraffin wax firstly, and three consecutive Sects. (5 μm) were stained with hematoxylin–eosin for analysis. Villus height and crypt depth were measured by using a microscope (LEICA RM2235, Olympus, Japan) at 40 × magnification. A total of 20 ~ 30 villus and their associated crypt per section were randomly selected, and villus length and crypt depth were analyzed by MShot Image Analysis System (MD50-T, Guangzhou, China).

### Real-Time PCR

Standard procedures of total RNA extraction and reverse transcription were conducted according to the instructions of RNA extraction kits and cDNA Synthesis SuperMix kit (TransGen Biotech, Beijing, China). Specific primer sequence for metallothionein was referred to the previous relevant study [23]; β-actin was selected as a housekeeping gene and then synthesized by Sangon Biotech (Shanghai, China). The results of qPCR were obtained by a particular detection system (Applied Biosystems, Foster City, USA) and the following protocol used was 3 min at 95 °C and then for 45 cycles of amplification (3 s at 95 °C and 30 s at 60 °C). Data were calculated using the 2^−(∆∆Ct)^ method [24].

### Statistical Analysis

Statistical analyses were performed by using the one-way ANOVA procedure of SAS software (SAS 8.1, Inst, Inc., Cary, NC), and multiple comparison was conducted using the Duncan method. If the data did not comply with the normal distribution or homogeneity, the Kruskal–Wallis test was used. Statistical significance was declared at *P* < 0.05 and trends at 0.05 < *P* < 0.10.

## Results

### Growth Performance

As shown in Table 3, compared with the control, ZL40 and ZS80 groups had a lower feed to gain ratio during 19 ~ 37 days (*P* < 0.05). However, the treatment did not affect the BW, ADG, ADFI, and feed to gain ratio (38 ~ 61 days) of the broilers. This suggested that supplementing ZL improved the feed efficiency of slow-growing broilers during the early period.

**Table 3:** Effects of zinc lactate supplementation on growth performance of broilers

Items		ZL40^1^	ZL60^1^	ZL80^1^	ZS80^2^	Control	SEM	*P*-value^3^
BW (g)	19 d	235.66	235.96	235.01	233.54	236.52	0.92	0.91
	38 d	839.54	822.74	806.92	802.09	804.86	7.05	0.44
	62 d	1793.79	1824.59	1752.58	1736.17	1799.02	21.37	0.73
ADG (g)	19 ~ 37 d	31.78	30.88	30.1	29.92	29.91	0.36	0.44
	38 ~ 61 d	39.76	41.74	39.4	38.92	41.42	0.67	0.67
	19 ~ 61 d	36.23	36.94	35.29	34.94	36.34	0.49	0.74
ADFI (g)	19 ~ 37 d	62.34	62.63	60.53	60.06	62.11	0.68	0.66
	38 ~ 61 d	104.87	105.35	101.28	99.77	104.96	1.01	0.27
	19 ~ 61 d	86.08	86.47	83.28	82.23	86.02	1.06	0.60
F/G	19 ~ 37 d	1.96^b^	2.03^ab^	2.01^ab^	2.00^b^	2.08^a^	0.01	0.047
	38 ~ 61 d	2.63	2.63	2.54	2.59	2.53	0.02	0.62
	19 ~ 61 d	2.37	2.33	2.36	2.35	2.36	0.01	0.83

^1^Supplemented with zinc lactate on the basal diet, and the additive dosage was 40 mg/kg, 60 mg/kg, 80 mg/kg, respectively; *ZL*, zinc lactate

^2^Supplemented with zinc sulfate on the basal diet, and the additive dosage was 80 mg/kg; *ZS*, zinc sulfate

^3^Data were obtained by one-way ANOVA, and difference significance was obtained by Duncan’s multiple comparisons (repetition *n* = 6)

### Slaughter Performance and Immune Organ Index

As shown in Tables 4 and 5, neither inclusion of the ZL nor the ZS in the broiler’s diet had an impact on the slaughter performance such as dressing percentage and immune organ index like thymus (*P* > 0.05).

**Table 4:** Effects of zinc lactate supplementation on slaughter performance and immune organ index of broilers

Items		ZL40^1^	ZL60^1^	ZL80^1^	ZS80^2^	Control	SEM	*P*-value^3^
Dressing percentage (%)	38 d	91.58	92.72	91.48	90.51	92.24	0.33	0.28
	62 d	90.15	91.03	91.58	90.13	91.23	0.29	1.00
Breast meat percentages (%)	38 d	13.04	14.11	14.14	15.70	14.35	0.38	0.37
	62 d	15.93	15.97	16.31	15.39	15.61	0.14	0.39
Thigh meat rate (%)	38 d	20.90	23.46	21.18	20.89	20.61	0.44	0.31
	62 d	22.06	23.03	21.65	22.14	22.37	0.32	0.19
Abdominal fat percentage (%)	38 d	3.00	2.67	2.98	2.88	3.18	0.26	0.99
	62 d	2.65	3.60	3.03	2.68	3.81	0.18	0.92
Percentage of eviscerated yield (%)	38 d	61.07	62.62	59.79	62.40	61.18	0.50	0.57
	62 d	67.24	66.95	67.67	65.87	67.22	0.37	0.88
Semi-eviscerated weight (%)	38 d	77.92	79.24	76.18	77.38	77.61	0.38	0.32
	62 d	81.92	81.56	82.68	81.35	82.36	0.31	0.93
Thymus (mg/g)	38 d	2.71	3.40	4.35	2.07	3.31	0.37	0.62
	62 d	2.03	2.62	2.30	2.18	2.07	0.13	0.83
Spleen (mg/g)	38 d	1.54	1.07	1.72	0.96	1.27	0.10	0.18
	62 d	1.39	1.33	1.28	1.32	1.28	0.08	0.69
Bursa of Fabricius (mg/g)	38 d	2.96	2.93	4.59	2.91	3.49	0.24	0.33
	62 d	0.80	1.39	1.30	0.95	0.90	0.09	0.67

^1^Supplemented with zinc lactate on the basal diet, and the additive dosage was 40 mg/kg, 60 mg/kg, 80 mg/kg, respectively; *ZL*, zinc lactate

^2^Supplemented with zinc sulfate on the basal diet, and the additive dosage was 80 mg/kg; *ZS*, zinc sulfate

^3^Data were obtained by one-way ANOVA, and difference significance was obtained by Duncan’s multiple comparisons (repetition *n* = 6)

**Table 5:** Effects of zinc lactate supplementation on intestinal morphology of broilers

Items	ZL40^1^	ZL60^1^	ZL80^1^	ZS80^2^	Control	SEM	*P*-value^3^
Duodenum							
Villus height (μm)	1720.48^A^	1485.40^BC^	1513.78^B^	1429.49^BC^	1327.87^C^	30.48	0.0005
Crypt depth (μm)	327.36	302.77	314.36	308.27	302.89	7.18	0.83
V/C	4.77	4.69	5.15	4.99	4.30	0.14	0.37
Jejunum							
Villus height (μm)	1236.59^BC^	1147.24^C^	1368.61^A^	1220.80^BC^	1302.43^AB^	20.09	0.0089
Crypt depth (μm)	365.34^A^	247.86^C^	264.34^BC^	202.53^D^	304.84^AB^	11.76	0.0002
V/C	3.59^D^	4.75^BC^	5.29^AB^	6.25^A^	4.15^CD^	0.22	0.0001
Ileum							
Villus height (μm)	1327.68^a^	1286.46^a^	1314.01^a^	1188.75^b^	1271.50^b^	15.35	0.02
Crypt depth (μm)	216.90	254.30	240.73	240.30	264.45	9.11	0.75
V/C	6.22	5.43	5.60	5.02	4.95	0.23	0.59

^1^Supplemented with zinc lactate on the basal diet, and the additive dosage was 40 mg/kg, 60 mg/kg, 80 mg/kg, respectively; *ZL*, zinc lactate

^2^Supplemented with zinc sulfate on the basal diet, and the additive dosage was 80 mg/kg; *ZS*, zinc sulfate

^3^Data were obtained by one-way ANOVA, and difference significance was obtained by Duncan’s multiple comparisons and that does not comply with the normal distribution or homogeneity; then, the significance was conducted by the Kruskal–Wallis test (repetition *n* = 6)

### Intestinal Morphology

No significant difference in the ratio of villus height to crypt depth in duodenum and ileum was observed. Compared with the control, villus height of duodenum was increased in treatment ZL40, and villus height of ileum was also enhanced in ZL40, ZL60, and ZL80 (*P* < 0.05). Villus height in the ZL80 group was higher than ZL40 and crypt depth in ZL80 was lower than ZL40. Hence, the villus height to crypt depth ratio in ZL80 was higher than ZL40. Moreover, the villus height to crypt depth ratio in ZL80 was also higher than that in control (*P* < 0.05). It was suggested that increasing the dietary dosage of ZL promoted the development of jejunum of broiler chickens. In addition, compared with the control, crypt depth in ZS80 decreased (*P* > 0.05) and villus height to crypt depth ratio increased (*P* < 0.05). These showed that both supplementations with 80 mg/kg ZL or 80 mg/kg ZS promoted the development of jejunum of broilers.

### Serum Biochemical Parameters

As shown in Table 6, compared with the control, the content of serum total protein at day 38 increased (*P* < 0.05) in ZL60 and serum albumin tended to be higher. Furthermore, compared with the control and ZS80, the activity of GSH-Px increased (*P* < 0.01) in ZL40, ZL60, and ZL80 at day 62, with ZL60 being the highest. Compared with the control, the activity of alkaline phosphatase in ZL80 and ZS80 tended to be higher (*P* = 0.053) than that in ZL40. These suggested that supplementation of 60 mg/kg ZL increased the content of serum total protein, albumin, and activity of GSH-Px.

**Table 6:** Effects of zinc lactate supplementation on serum biochemical indexes of broilers

Items		ZL40^1^	ZL60^1^	ZL80^1^	ZS80^2^	Control	SEM	*P*-value^3^
Total protein (g/l)	38 d	35.74^ab^	39.74^a^	35.30^b^	33.12^b^	34.91^b^	0.68	0.04
	62 d	38.73	37.9	38.03	38.93	37.68	0.62	0.97
Albumin (g/l)	38 d	15.71^ab^	17.30^a^	16.37^ab^	15.03^b^	15.28^b^	0.28	0.07
	62 d	16.17	16.19	15.55	16.4	16.48	0.21	0.68
Urea (mmol/l)	38 d	0.61	0.42	0.48	0.38	0.34	0.03	0.12
	62 d	0.36	0.36	0.32	0.34	0.31	0.01	0.47
Cholesterol (mmol/l)	38 d	3.83	4.69	4.15	3.84	3.93	0.15	0.33
	62 d	3.71	4.35	3.90	4.02	4.13	0.09	0.22
Alkaline phosphatase (U/l)	38 d	4018.47	4355.23	3440.71	3727.32	3613.36	286.51	0.90
	62 d	1640.52^b^	2136.68^ab^	2930.26^a^	3066.87^a^	2232.46^ab^	168.93	0.053
MDA (nmol/mL)	38 d	3.55	4.06	3.75	3.33^ab^	3.19	0.09	0.10
	62 d	3.30	3.04	2.92	2.81	3.39	0.09	0.27
GSH-PX (U/ml)	38 d	674.76	639.96	632.52	611.40	605.04	9.41	0.19
	62 d	493.29^B^	517.43^A^	495.04^B^	470.78^C^	459.38^C^	3.46	< .0001
SOD (U/ml)	38 d	210.67	200.56	186.29	188.42	201.83	4.32	0.45
	62 d	228.38	236.18	259.98	236.18	251.06	5.32	0.41

^1^Supplemented with zinc lactate on the basal diet, and the additive dosage was 40 mg/kg, 60 mg/kg, 80 mg/kg, respectively; *ZL*, zinc lactate

^2^Supplemented with zinc sulfate on the basal diet, and the additive dosage was 80 mg/kg; *ZS*, zinc sulfate

^3^Data were obtained by one-way ANOVA, and difference significance was obtained by Duncan’s multiple comparisons and that does not comply with the normal distribution or homogeneity; then, the significance was conducted by the Kruskal–Wallis test (repetition *n* = 6)

### Gene Expression of Hepatic Metallothionein

**Fig. 1:**
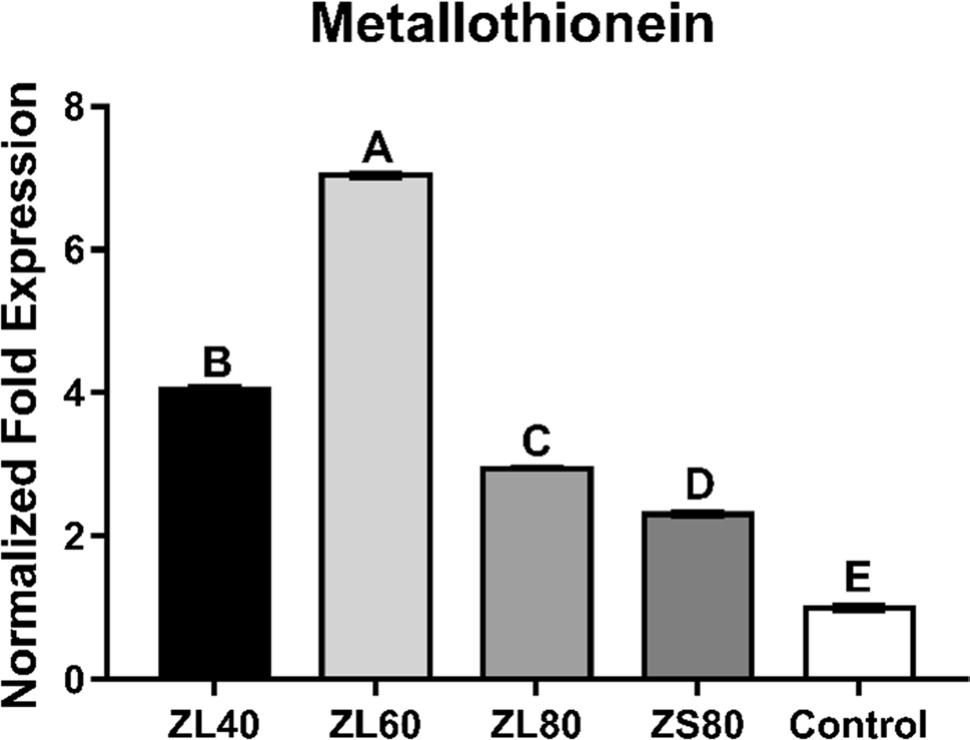
Effects of zinc lactate supplementation on metallothionein mRNA expression in the liver. ZL40, ZL60, ZL80: supplemented with zinc lactate on the basal diet, and the additive dosage was 40 mg/kg, 60 mg/kg, 80 mg/kg, respectively. ZS80: supplemented with zinc sulfate on the basal diet, and the additive dosage was 80 mg/kg. The different capital letters on the columns indicated a significant difference. Data were obtained by one-way ANOVA, and difference significance was obtained by Duncan’s multiple comparisons and that does not comply with the normal distribution or homogeneity; then, the significance was conducted by the Kruskal–Wallis test (repetition *n *= 6)

As shown in Fig. 1, the metallothionein mRNA expression in the liver was affected by treatment (*P* < 0.01). Compared with the control, ZL80 and ZS80 both promoted (*P* < 0.01) the metallothionein mRNA expression. It is interesting that the relative expression level of metallothionein in ZL80 was higher than that in ZS80 (*P* < 0.01). Compared with the control, the mRNA expression of metallothionein in ZL40, ZL60, and ZL80 increased (*P* < 0.01). Moreover, the ZL60 group had the highest value (*P* < 0.01). The above results suggested that either inclusion of ZL or ZS in the broiler’s diet promoted the hepatic metallothionein mRNA expression and dietary supplementation of ZL at 60 mg/kg had the best results in this study.

## Discussion

Promoting the growth of animals and reducing the feed conversion rate has been one of the objectives in animal nutrition due to the worldwide food shortage. Hence, growth performance is normally considered as a basic indicator to evaluate the nutritional requirements of broiler and is highly valued by producers. In the present study, the addition of 40 mg/kg zinc lactate and 80 mg/kg zinc sulfate in broiler diet lowered the feed to gain ratio during the early period without affecting the ADG and ADFI, which is partly similar to the previous reports [25–27]. A previous study indicated that the weight gain of fast-growing broilers was less sensitive to the zinc levels if the zinc requirement of growth is met [28]. Hence, in terms of slow-growing broilers, a corn-soybean meal diet might be supplied sufficient Zn for their growth. A study has reported that ADG, ADFI, and feed conversion ratio of broiler chickens (Ross 308) were not affected by dietary organic zinc supplementation (60 mg/kg and 120 mg/kg) [29], which was similar to ours. However, whether dietary organic zinc supplementation has an impact on the feed conversion ratio of slow-growing broiler chicks needed more research to confirm. It is interesting to note that the feed conversion ratio of broiler chickens (Cobb 500) was improved by the interaction of zinc sources and levels [30]. This suggested that a blend of dietary organic zinc and inorganic zinc may have a synergistic promoting effect on the growth performance of broiler chickens. Recently, a study showed that zinc supplementation could increase the nutrient digestibility of mink [31]. Therefore, in the current study, the improved feed efficiency might be partly attributed to the effect of zinc lactate or zinc sulfate on the digestion, absorption, and metabolism of broilers. However, the digestibility of nutrients was not affected by sources of zinc supplementation in pigs [12]. This would require more attention in the following broiler chicken studies.

Intestinal villus (the sites of nutrient absorption) and the crypt depth (indicate the turnover rate of renewing villus) are playing a crucial role in digestive systems. Nutrient absorption ability is often evaluated by the ratio of villi height to crypt depth in animals. Zinc could promote cell proliferation and protein synthesis in the crypt base [32]. Results of the current study demonstrated that zinc lactate increased the villus height of the duodenum and ileum. Supplementation with 80 mg/kg zinc lactate and 80 mg/kg zinc sulfate also decreased the crypt depth of jejunum of broilers. Dietary zinc could improve intestinal morphology by increasing villi height or reducing crypt depth in different kinds of animals [33–35]. Previous studies showed that inorganic zinc (zinc sulfate) was more efficient in improving the villi height of jejunum compared with organic zinc (zinc glycine) [36], which was contrary to our results that villi height of jejunum in ZL80 was higher than ZS80. A recent study showed that zinc supplied in a feed as an amino acid complex (60 mg/kg) improved the intestinal morphology with an increased villus height and the ratio of villus height to crypt depth in the duodenum [37]. This suggested that organic zinc supplementation had a better effect than inorganic zinc. The inconsistent results may be attributed to the form of organic zinc supplied and needs to be determined by the subsequent studies.

The main physiological role of serum total protein is to maintain the colloid osmotic pressure. It can reflect the hepatic protein synthesis ability and often was considered an important indicator of liver damage [38]. Both levels of serum albumin and total protein are closely related to the body’s nutritional status and protein metabolism. Then, a higher level of serum total protein indicates an exuberant hepatic protein metabolism, which might be beneficial to the growth of animals. Previous research demonstrated that zinc glycine (organic zinc) increased the content of serum total protein [34]. In the current study, the inclusion of 60 mg/kg zinc lactate increased the content of total protein and albumin. These suggested that zinc lactate was beneficial to the protein synthesis in the liver of the broiler which might help to alleviate liver damage. Metallothionein plays an important role in the detoxification of heavy metals [39]. The increasing concentration of metallothionein can be also beneficial to liver function. Current results showed that supplementation with zinc lactate (40 ~ 80 mg/kg) and zinc sulfate (80 mg/kg) in diet promoted the metallothionein gene expression, which was in accordance with Min et al. (2019) [13]. Furthermore, the content of metallothionein could be a crucial indicator of the zinc status of the body and suitable for analysis of bioavailability [28, 40]. Interestingly, the content of metallothionein in ZL40, ZL60, and ZL80 groups was higher than in ZS80 and control. It proved zinc lactate has a higher bioavailability than inorganic zinc and thus more beneficial to liver health. Zn can bind to the metal reactive element transcription factor, which could recognize and initiate the transcription of metallothionein [4, 41, 42]. This may be the reason why the content of metallothionein in tissues can be used as an indicator of zinc status [43].

Zn is an important component of various enzymes involved in multiple biochemical metabolic processes [4]. Alkaline phosphatase is often used to evaluate the body utilization of Zn and increased activity of alkaline phosphatase refers to a higher utilization rate of Zn. In the present study, compared with the control, the activity of alkaline phosphatase in ZL80 and ZS80 increased by 31.26% and 37.37% (*P* > 0.05), respectively. Combined with the increased hepatic metallothionein content, these results suggested that supplementing zinc lactate had a higher bioavailability than zinc sulfate which was consistent with previous research [6]. Content of zinc in tibia ash was used to evaluate the bioavailability of zinc in a previous study [6]. Unfortunately, we did not collect the tibia samples. Zinc is a key component of some antioxidant enzymes like SOD [12, 44] and its beneficial effects on oxidative stress have been widely reported [44–46]. Some previous studies indicated that zinc methionine (organic zinc) increased the activities of serum SOD and GSH-Px of broilers [17, 47]. This was partly consistent with our current study that zinc lactate increased the activity of serum GSH-Px compared with ZS80 and the control group. This may derive from the increased concentration of active GSH-Px [48]. It suggested zinc lactate is more beneficial to alleviate oxidative stress, thus contributing to animals’ health. Dietary Zn did not change the activity of liver SOD [28] and it was consistent with our current results. Additionally, what calls for special attention is that zinc could inhibit GSH-Px activity and induce oxidative stress at a high level [49–52]. Few changes in oxidative stress parameters were observed on the broilers in the ZL group. Therefore, it would be interesting to investigate whether supplementation of ZL would exert a beneficial impact on the antioxidant status under challenging conditions such as under a high ambient temperature condition.

In summary, supplementing ZL could decrease the feed to gain ratio and improve small intestinal morphology by increasing the villus height and the ratio of villus height to crypt depth. It can also increase the concentration of serum total protein and albumin as well as the activity of GSH-Px and the content of hepatic metallothionein. The possible reason could be attributed to its higher bioavailability, especially with the dosage at 60 mg/kg.

## Data Availability

The data used to support the findings are all included in the article.
